# Evidence-based clinical standard for the diagnosis and treatment of invasive lung aspergillosis in the patient with oncohematologic disease

**DOI:** 10.1016/j.bjid.2025.104517

**Published:** 2025-02-24

**Authors:** Jorge Alberto Cortés, Diego Andrés Rodríguez-Lugo, Martha Carolina Valderrama-Rios, Ricardo Rabagliati, Domenico Capone, Carlos Arturo Álvarez-Moreno, Fabio Varón-Vega, Laura Cristina Nocua-Báez, Cándida Diaz-Brochero, Leonardo Enciso Olivera, Sonia Isabel Cuervo-Maldonado, Luis Thompson, Dora E. Corzo-León, Luis E. Cuéllar, Erika Paola Vergara, Fernando Riera, Patricia Cornejo-Juárez, Rita Rojas, Beatriz L. Gómez, Adriana Marcela Celis-Ramírez, José Luis Sandoval-Gutiérrez, Mauricio Sarmiento, Diana Lorena Ochoa, Marcio Nucci

**Affiliations:** a Universidad Nacional de Colombia, Facultad de Medicina, Departamento de Medicina Interna, Bogotá, Colombia; b Hospital Universitario Nacional de Colombia, Unidad de Infectología, Bogotá, Colombia; c Pontificia Universidad Católica de Chile, Escuela de Medicina, Departamento de Enfermedades Infecciosas del Adulto, Santiago, Chile; d Instituto de Doenças do Tórax da Universidade Federal do Rio de Janeiro (UFRJ), Rio de Janeiro, RJ, Brazil; e Disciplina de Pneumologia da Universidade do Estado do Rio de Janeiro (UERJ), Rio de Janeiro, RJ, Brazil; f Clínica Universitaria Colombia, Clínica Colsanitas Grupo Keralty, Bogotá, Colombia; g Medicina Interna-Neumología-Cuidado Intensivo, Unidad de Cuidado Intensivo Médica, Fundación Neumológica Colombiana, Fundación Cardioinfantil, Bogotá, Colombia; h Pontificia Universidad Javeriana, Hospital Universitario San Ignacio, Departamento de Medicina Interna, Bogotá, Colombia; i Hospital Universitario Nacional de Colombia, Unidad de Hematología, Bogotá, Colombia; j Instituto Nacional de Cancerología, Grupo Infectología, Bogotá, Colombia; k Unidad de Infectología, Clínica Alemana ‒ Universidad del Desarrollo, Departamento de Medicina Interna, Santiago, Chile; l Universidad de Exeter, Centro de Micología Médica Del Medical Research Council, Exeter, United Kingdom; m Instituto Nacional de Enfermedades Neoplásicas, Unidad de Infectología, Lima, Perú; n Universidad Nacional Federico Villarreal, Lima, Perú; o Hospital Infantil Universitario de San José, Departamento de Infectología, Bogotá, Colombia; p División de Enfermedades Infecciosas, Sanatorio Allende Córdoba, Córdoba, Argentina; q Enfermedades Infecciosas, Universidad Nacional de Córdoba, Córdoba, Argentina; r Instituto Nacional de Cancerología, Departamento de Infectología, Ciudad de México, México; s Hospital General Plaza de la Salud, Santo Domingo, República Dominicana; t Grupo de Estudios en Microbiología Traslacional y Enfermedades Emergentes (MICROS), Escuela de Medicina y Ciencias de la Salud, Universidad del Rosario, Bogotá, Colombia; u Grupo de Investigación Celular y Molecular de Microorganismos Patógenos (CeMoP), Departamento de Ciencias Biológicas, Universidad de los Andes, Bogotá, Colombia; v Laboratorio de Investigación Celular y Molecular de Patógenos (CeMoP), Departamento de Ciencias Biológicas, Universidad de los Andes, Bogotá, Colombia; w Instituto Nacional de Enfermedades Respiratorias, Ciudad de México, México; x Departamento de Hematología y Oncología, Escuela de Medicina, Pontificia Universidad Católica de Chile, Santiago, Chile; y Departamento de Radiología e Imágenes Diagnosticas, Hospital Universitário Nacional de Colombia, Bogotá, Colombia; z Departament of Internal Medicine, Hospital Universitario, Universidade Federal do Rio de Janeiro, Río de Janeiro, RJ, Brazil; aa Grupo Oncoclínicas, Brazil

**Keywords:** Invasive pulmonary aspergillosis, Hematologic neoplasms, Antifungal agents, Latin America

## Abstract

Aspergillosis is a disease caused by the filamentous fungus *Aspergillus* spp. with a spectrum of clinical presentation that includes invasive and noninvasive forms. The invasive clinical presentation of aspergillosis most frequently affects people with compromised immune systems. In patients with oncohematologic pathology, invasive lung aspergillosis is a significant opportunistic mycosis, because it occurs frequently and has a major impact on morbidity, mortality, and high costs. The global problem of antimicrobial resistance, to which improper use of antifungals contributes, has put *Aspergilus* spp. in the spotlight, so it is important to generate guidelines for guidance in the proper use of antifungals in the management of invasive lung aspergillosis, to obtain better clinical outcomes and promote rational use of antifungals. This guideline contains recommendations for diagnosing and treating invasive lung aspergillosis in patients with oncohematologic disease, based on evidence and defined through a participatory process of expert consensus, for the Latin American context.

## Introduction

The invasive aspergillosis typically affects immunocompromised patients and is one of the leading causes of death from infections in patients with oncohematologic pathologies. The main *Aspergillus* species that cause this disease are *A. fumigatus, A. flavus, A. terreus*, and A*. niger*.^1^^,^^2^ The number of aspergillosis cases increases every year, which has been attributed to the growing number of immunocompromised individuals, optimization in diagnosis, improved cancer treatments, and the development of new immunosuppressive drugs.^3^

Several oncohematological pathologies carry a high-risk of aspergillosis, equal to or higher than 10 %, including acute myeloid leukemia, myelodysplastic syndrome (in induction therapy), and allogeneic hematopoietic stem cell transplantation, especially when associated with graft-versus-host disease.^4^^,^^5^ Although with data that may vary between centers, in general, autologous hematopoietic stem cell transplant recipients, patients with acute lymphoid leukemia, and those with Hodgkin's or nonHodgkin's lymphomas are considered to be associated with a low risk of aspergillosis,^1^^,^^6^ although, of course, this risk can vary depending on factors like pathology, chemotherapy regimen, hospital care setting, and timing of patient evaluation.

Globally, it is estimated that >300,000 cases occur per year, with mortality data in some registries exceeding 90 %. *Aspergillus* spp. is the main cause of invasive fungal disease in patients with allogeneic hematopoietic stem cell transplantation in Latin America, making up 63.4 % of reported cases in the region,^7^^,^^8^ with two peak times of presentation, within the first 30-days and after 90-days posttransplant, affecting 3 % of patients with this type of transplantation, the most associated species being *A. fumigatus* and the main organ involved being the lung.^9^^,^^10^ In addition to the constant increase in cases of this entity, an increasing number of cases with identification of azole-resistant *A. fumigatus* has also been observed, which are associated with a mortality 20 %‒30 % higher than in cases of infection by sensitive strains^11^, ^12^, ^13^ with reported mortality rates ranging from 47 % to 100 %. In 2022, the World Health Organization (WHO) classified *A. fumigatus* as a critical priority pathogen to guide research, development, and public health actions.^14^

Restricted access to advanced diagnostic tools is one of the reasons why invasive aspergillosis, among other fungal infections, is underdiagnosed in low- and middle-income countries,^15^ Latin America is a clear example of this. Falci and Pasqualotto, in a study conducted in several countries in the region found that, in 107 laboratories evaluated, galactomannan was available in only 48 %, with even more worrisome deficiencies for Polymerase Chain Reaction (PCR), which was found in only 20 % of 73 institutions with access to molecular testing. In this same study, regarding the availability of antifungals evaluated in 124 centers, access to liposomal amphotericin B and voriconazole was found in only 49 % and 55 %, respectively.^16^

Ortiz et al., in a study done in 2023, evaluated the diagnostic and therapeutic capabilities for the management of invasive fungal infections in Honduras through a survey of microbiologists and clinicians, finding that only 2.6 % of the institutions evaluated had antigen detection available for Aspergillus spp. infections and that PCR was not available in any center. In addition, only 21.4 % and 35.7 % of the institutions had availability of liposomal amphotericin B and voriconazole, respectively.^17^

Antifungal resistance is an increasingly reported problem in Latin America, with descriptions in clinical and environmental samples in Argentina, Brazil, Peru, Colombia,^12^ and Cuba^18^; however, in the work of Falci and Pasqualotto, only 14 % of laboratories had antifungal susceptibility testing for molds.^16^

Therefore, guidelines for diagnosing and treating invasive lung aspergillosis in patients with oncohematological disease, are needed to provide quality health care, improve clinical outcomes, and promote responsible antifungal medications.

### Scope

This Evidence-Based Clinical Standard (EBCS) is intended for healthcare personnel caring for adult patients (over 18-years of age) with oncohematologic disease, and for decision-makers or those involved in generating health policies in healthcare institutions in Latin America.

### Target

To develop recommendations and algorithms for diagnosing and treating invasive lung aspergillosis in patients with oncohematologic disease systematically and collaboratively using the best available evidence.

### Patients considered and clinical aspects addressed

The clinical practice recommendations and algorithms in this EBCS are for adult patients (18-years old or older) with acute hematologic malignancy or those who have had hematopoietic stem cell transplants, who are asymptomatic or clinically suspected or confirmed diagnosis of invasive lung aspergillosis, and address interventions for prophylaxis, diagnosis, and treatment.

### Patients not considered and clinical issues not addressed

This EBCS excludes the following patient groups: children or adolescents (under 18-years-old), pregnant women, adults living with human immunodeficiency virus, patients with a diagnosis or history of solid neoplasm, solid organ transplant recipients, patients with other immunosuppressive conditions or medications, apparently immunocompetent patients, or patients in an intensive care unit with coinfection (e.g., patients with influenza or SARS-CoV-2 infection). The EBCS does not address interventions for prophylaxis, diagnosis, or treatment of aspergillosis in other anatomic locations (e.g., sinuses, central nervous system, bone [i.e., osteomyelitis, septic arthritis]), heart [i.e., endocarditis, myocarditis, pericarditis], skin, etc.).

### Users to whom the clinical practice guide is directed and healthcare field

The recommendations and clinical practice algorithms in this EBCS are for health professionals involved in caring for adult patients with acute hematologic neoplasia or those who have had hematopoietic progenitor transplants. This includes specialists in internal medicine, hematology, oncohematology, pulmonology, infectious diseases, critical medicine, and intensive care, as well as nurse practitioners, pharmaceutical chemists, clinical laboratory personnel, and others involved in the care process of this population group.

## Methods

The clinical practice recommendations and algorithms were developed through a process led by the National University Hospital of Colombia, in collaboration with the Clinical Research Institute of the National University of Colombia and the National University of Colombia. This process, called Evidence-Based Clinical Standards,^19^ consists of six phases carried out sequentially: 1) EBCS development group composition; 2) Definition EBCS’ scope and objective; 3) Systematic search for Clinical Practice Guidelines (CPG); 4) Screening, quality evaluation, and selection of CPG; 5) Elaboration of preliminary recommendations and algorithms; 5a) Elaboration a comparative table of evidence; 5b) Review and discussion of recommendations and algorithms by the development group; 6) Final elaboration of recommendations and algorithms; 6a) Review and discussion of recommendations and algorithms in a participatory process with expert consensus.

### EBCS development group composition

The EBCS development group consisted of eleven members, including thematic and methodological experts: physicians with training in internal medicine, infectious diseases, pulmonology, oncohematology, and clinical epidemiology, with experience in systematic literature reviews, synthesis and qualification of evidence, and participatory processes (JAC, DAR, MCV, MN, RMR, DC, CAA, FV, LCN, CDB, LE). Prior to the start of activities, each development group member filled out a conflict-of-interest form. If a conflict was declared, it was reviewed to determine how it might affect their participation.

### Definition of scope and objective

The scope and objective of the EBCS were defined by answering key questions: Why is it being done? Is there variability in current practice? What is it being done for? Who is it intended for? Who will use it?^20^ These guided the final formulation of the scope, objective, target patient group, clinical aspects to be addressed, and user population and care setting to which the content of the EBCS is addressed.

### Systematic search of CPG

Systematic searches were done to identify CPGs that corresponded to the proposed scope and objective, published between 2014 and 2023, regardless of language.

Highly sensitive electronic search strategies were designed. The search was conducted from 6 to July 9, 2023 on the websites of the following CPG compiling and developing bodies: Guidelines International Network (GIN), Agency for Healthcare Research and Quality/National Guidelines Clearinghouse (AHRQ), CMA Infobase: Clinical Practice Guidelines, Catalog of Clinical Practice Guidelines in the National Health System, National Institute for Clinical Excellence (NICE), Scottish Intercollegiate Guidelines Network (SIGN), and WHO, and in the Medline and Embase databases using search strategies adapted for each search engine using Boolean, truncation and proximity operators, free text terms, and controlled vocabulary, using key terms such as “Aspergillus”, “Aspergillosis”, “Pulmonary Aspergillosis”, “Hematologic Neoplasms”, “Hematopoietic Stem Cell Transplantation”, and “Bone Marrow Transplantation”. More information on search strategies is presented in the Supplementary Material Table 1.

### Screening, quality assessment, and selection of CPGs

Once the search results were obtained, two reviewers (DAR, LCN) independently screened and made the primary selection of references by title and abstract, selecting the references corresponding to CPGs, expert consensus, or generation of recommendations that addressed the aspects defined in the scope and objective of this guideline. Subsequently, two reviewers (DAR, LCN) independently performed the screening and secondary selection of the full text of the selected references, using the criteria from the modified tool 7 of the Methodological Guide for the adoption-adaptation of evidence-based clinical practice guidelines of the Ministry of Health and Social Protection of Colombia^21^: CPG with generation of evidence-based recommendations, CPG with a development process and conformation of a developer group, CPG with a reliable evidence search, date of the last update of the search, and use of the GRADE (Grading of Recommendations, Assessment, Development, and Evaluation) system for the global grading of evidence (Table 1). References without full text access were discarded. Discrepancies between the two reviewers were resolved through review, discussion, and consensus, or by involving a third reviewer if needed.

**Table 1:** Overall grading of evidence using the GRADE system.

**Levels of certainty of evidence**^27^	
High	There is high confidence that the true effect is close to the effect estimate.
Moderate	The confidence in the effect estimate is moderate. The true effect may be close to the estimate, but it could be substantially different.
Download	Confidence in the effect estimate is limited. The true effect may be substantially different from the effect estimate.
Very low	The confidence in the effect estimate is very low. The true effect is likely to be substantially different from the effect estimate.
**Meaning of the strength and direction of the recommendations**^27^	
Strongly in favor	The benefits of the intervention clearly outweigh the undesirable effects.
Conditionally in favor	The benefits of the intervention probably outweigh the undesirable effects.
Strongly against	The undesirable effects of the intervention clearly outweigh the benefits.
Conditionally against	The undesirable effects of the intervention probably outweigh the benefits.
Implications of the strength of the recommendation^28^	
**The implications of a strong recommendation are**	
For patients	Most people in this situation would desire the recommended course of action, and only a small proportion would not.
For clinicians	Most patients should receive the recommended course of action.
For policy makers	The recommendation can be adopted as a policy in most situations.
**The implications of a conditional recommendation are**	
For patients	Most people in this situation would like the recommended course of action, but many do not.
For clinicians	You should recognize that different options will be appropriate for different patients, and you should help each patient arrive at a management decision consistent with his or her values and preferences.
For policy makers	Policy formulation will require substantial debate and the involvement of many stakeholders.

The CPGs selected after the screening were evaluated for quality by the development group using the AGREE II tool.^22^ Each guideline was evaluated independently by three reviewers, including a clinical expert and a methodological expert. If any necessary information was identified, additional details were requested from the guideline developers via email. As a result of the quality assessment, CPGs that scored 60 % or higher in methodological rigor and editorial independence domains were identified and selected.

Using the methodology described, five references were selected, including the CPG developed by the Infectious Diseases Society of America (IDSA): “Practice Guidelines for the Diagnosis and Management of Aspergillosis: 2016 Update by the Infectious Diseases Society of America”, published in 2018;^23^ chapters 4 and 7 of the Australian and New Zealand consensus for the treatment of invasive fungal diseases and the use of antifungal agents in the hematology/oncology setting, published in 2021; “Consensus guidelines for antifungal prophylaxis in hematological malignancy and haemopoietic stem cell transplantation, 2021″;^24^ and “Consensus guidelines for the diagnosis and management of invasive aspergillosis, 2021″;^25^ and the two sections of the Colombian consensus, published in 2022: “Section 1. Colombian consensus on the diagnosis and follow-up of invasive aspergillosis and Aspergillus disease in adult and pediatric patients”^6^ and “Section 2. Colombian consensus for prophylaxis, treatment and prevention of invasive aspergillosis in adult and pediatric patients”.^26^ The CPG screening and selection process is summarized in the PRISMA diagram (Supplementary Material Fig. 1).

### Preparation of proposed recommendations and preliminary algorithm

A comparative table was prepared based on the reading and extraction of information from the five selected CPGs for the preparation of the proposed recommendations and preliminary algorithm. This table included recommendations for each previously defined clinical aspects to be addressed in the EBCS, along with the evidence certainty and recommendation strength. Informal virtual consensus meetings, lasting approximately 2 h each, were held with all development group members. They presented and reviewed the information from the comparative table and discussed and constructed the proposed recommendations and preliminary algorithm of the EBCS.

### Expert consensus

Finally, the proposed recommendations and preliminary algorithm were reviewed in an expert consensus by professionals from Argentina (FR), Brazil (MN, DC), Chile (RMR, LT, MS), Colombia (JAC, DAR, DAR, CAA, FV, LCN, CDB, LE, SIC, EPV, BLG, AMCR, DLO), Mexico (DEC, PC, JLS), Peru (LEC), and Dominican Republic (RAR). These professionals, trained in infectious diseases (JAC, RMR, CAA, LCN, SIC, LT, DEC, LEC, EPV, FR, PC, RAR, MN), microbiology (BLG, AMCR), diagnostic imaging (DLO), pulmonology (DC, FV, JLS), oncohematology (LE, MS), and internal medicine (CDB, DAR), provided input from various specialties involved in diagnosing and treating invasive lung aspergillosis in patients with oncohematologic disease in Latin America. Each consensus participant completed a conflict-of-interest form. Any declared conflicts were analyzed to determine their impact on participation.

Two virtual consensus meetings, each lasting approximately 4 h, were held. During the meetings, the proposed recommendations and preliminary algorithm were presented and reviewed, and using a real-time Delphi methodology, the formulation and construction of the recommendations and final algorithm were carried out. Voting was carried out anonymously and electronically, to evaluate the degree of agreement with each recommendation and section of the algorithm. A Likert scale from 1 to 9 was used, where 1 corresponded to strongly disagree, 5 to neither agree nor disagree, and 9 to completely agree.^29^ Agreement was defined as agreement when ≥ 60 % of the votes were in the 4–9 range and < 20 % of the votes were in the 4–6 range of the scale. No agreement (i.e., no consensus) was considered when ≥ 40 % of the votes were in the range of 1–3. If no consensus was considered in the first round, a discussion and a new round of voting were held, with a maximum of three rounds allowed for each question.

## Results

### Flowchart

**Fig. 1:**
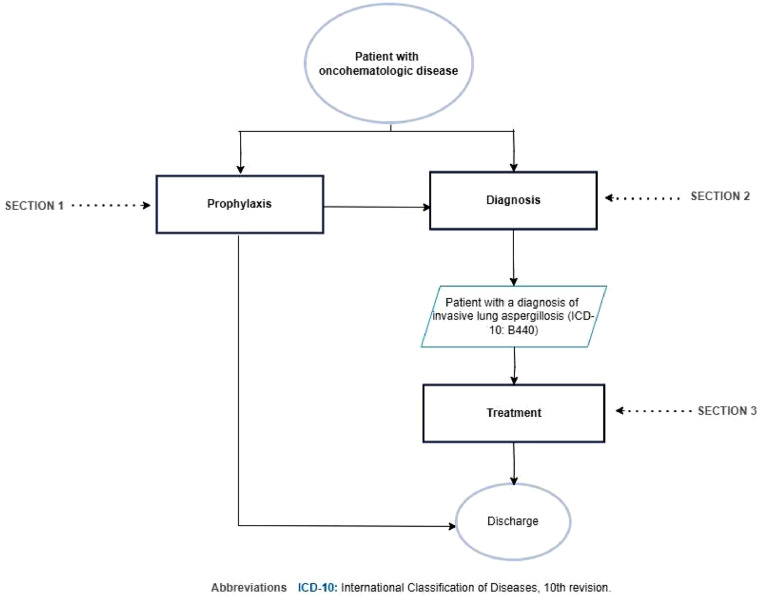
Flowchart for the diagnosis and treatment of invasive lung aspergillosis in the patient with oncohematologic disease. ICD-10, International Classification of Diseases, 10th revision.

Fig. 1 shows the flowchart for the diagnosis and treatment of invasive lung aspergillosis in the patient with oncohematologic disease.

**Fig. 2:**
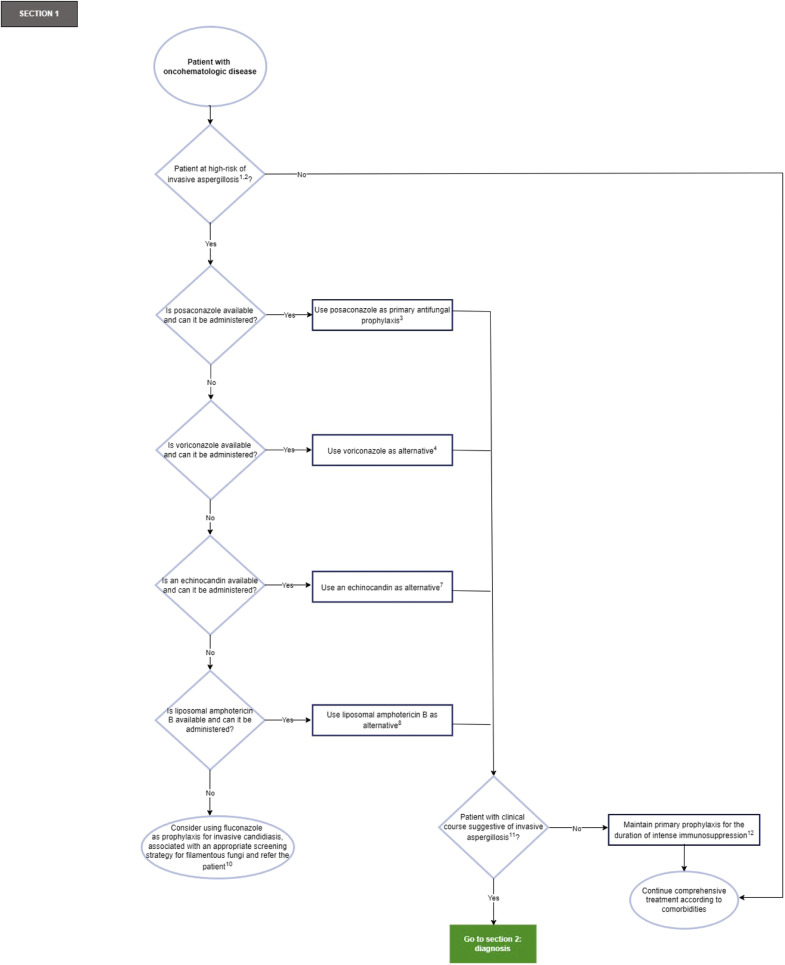
Flowchart Section 1: Primary antifungal prophylaxis of the patient with oncohematologic disease at high-risk of invasive lung aspergillosis.

Fig. 2 shows Section 1 of the flowchart (primary antifungal prophylaxis of the patient with oncohematologic disease at high-risk of invasive lung aspergillosis).

***Recommendations flowchart Section 1:*** Primary antifungal prophylaxis of the patient with oncohematologic disease at high-risk of invasive lung aspergillosis.

1) Primary antifungal prophylaxis is recommended for patients with the following conditions (Strong recommendation, high-quality evidence, GRADE)^26^:
  • Severe graft-versus-host disease.
  • Acute myeloid leukemia or myelodysplastic syndromes in induction with intensive chemotherapy.
  It is recommended to individualize the decision to initiate primary antifungal prophylaxis in patients with the following conditions (expert recommendation):^24^, ^26^
  • Administration of corticosteroid doses equivalent to >1 mg/kg of prednisolone and neutrophils less than 1 × 10^9^/L for >1-week.
  • Administration of corticosteroid doses equivalent to >2 mg/kg of prednisolone for >2-weeks.
  • Neutrophils <0.1 × 10^9^/L for >3-weeks, or <0.5 × 10^9^/L for >5-weeks.
  • Allogeneic transplantation of unrelated, mismatched hematopoietic stem cells or cord blood.
  • Acute lymphoblastic leukemia in induction/reinduction.
2) Due to the absence of high-level evidence, the routine use of primary antifungal prophylaxis is not recommended for most patients with oncohematologic disease on therapy undergoing new hematologic therapies. The decision to initiate primary antifungal prophylaxis should be individualized for each patient (expert recommendation).^24^
3) Posaconazole is recommended as primary antifungal prophylaxis for patients at high-risk of invasive lung aspergillosis (strong recommendation, high-quality evidence, GRADE)^23^^,^^24^^,^^26^ (Table 2).

**Table 2:** Primary antifungal prophylaxis of the patient with oncohematological disease at high-risk of invasive lung aspergillosis.

**Antifungal**	**Dosage**
Posaconazole	Tablet: 300 mg every 12 h on the 1st day and then 300 mg everyday PO
	Solution: 200 mg every 8 h PO
Voriconazole	4 mg/kg every 12 h PO or IV
Caspofungin	70 mg on the 1st day then 50 mg every day IV
Micafungin	100–150 mg every day IV
Liposomal amphotericin B	3 mg/kg 3-times a week IV

PO, Per Oral; IV, Intravenous.

Taken and adapted from.^23^^,^^24^^,^^30^.
4) Voriconazole is recommended as an alternative for primary antifungal prophylaxis if posaconazole is contraindicated and/or not possible (Strong recommendation, moderate-quality evidence, GRADE)^23^^,^^24^ (Table 2).
5) The expert panel considers that there is insufficient evidence to recommend itraconazole-SUBA (SUper BioAvailability) as primary antifungal prophylaxis for patients at high-risk of invasive lung aspergillosis.
6) The expert panel considers that there is insufficient evidence to recommend isavuconazole as primary antifungal prophylaxis for patients at high-risk for invasive lung aspergillosis.
7) An echinocandin, preferably caspofungin, is recommended as an alternative for primary antifungal prophylaxis if azoles are contraindicated and/or not possible. The use of micafungin could be considered (strong recommendation, high-quality evidence, GRADE)^26^ (Table 2).
8) Liposomal amphotericin B is recommended as an alternative for primary antifungal prophylaxis if echinocandins are contraindicated and/or not possible (conditional recommendation, moderate-quality evidence, GRADE)^24^ (Table 2).
9) The expert panel considers that there is insufficient evidence to recommend nebulized amphotericin B as primary antifungal prophylaxis for patients at high-risk for invasive lung aspergillosis.
10) Good practice point: If no other primary antifungal prophylaxis options are available for filamentous fungi, consider using fluconazole (400 mg/day orally) as prophylaxis for invasive candidiasis, associated with an appropriate screening strategy for filamentous fungi, and refer the patient to a center where antifungal prophylaxis against filamentous fungi options are available.
11) In cases of suspected gap infection, verify adherence to antifungal prophylaxis, check for possible drug interactions, and other relevant factors (strong recommendation, high-quality evidence, GRADE),^25^ and the diagnostic procedure for gap infection should be initiated.
12) It is recommended to maintain primary antifungal prophylaxis for the duration of intense immunosuppression (strong recommendation, high-quality evidence, GRADE).^23^^,^^26^ The decision to discontinue antifungal prophylaxis should be individualized, taking into account neutrophil recovery (> 0.5 × 10^9^/L) and tapering of the dose of immunosuppressive drugs used in graft-versus-host disease.^23^^,^^26^

Good practice point: If primary antifungal prophylaxis was started with an antifungal other than an azole, consider switching to an extended-spectrum azole for continued prophylaxis.

**Fig. 3:**
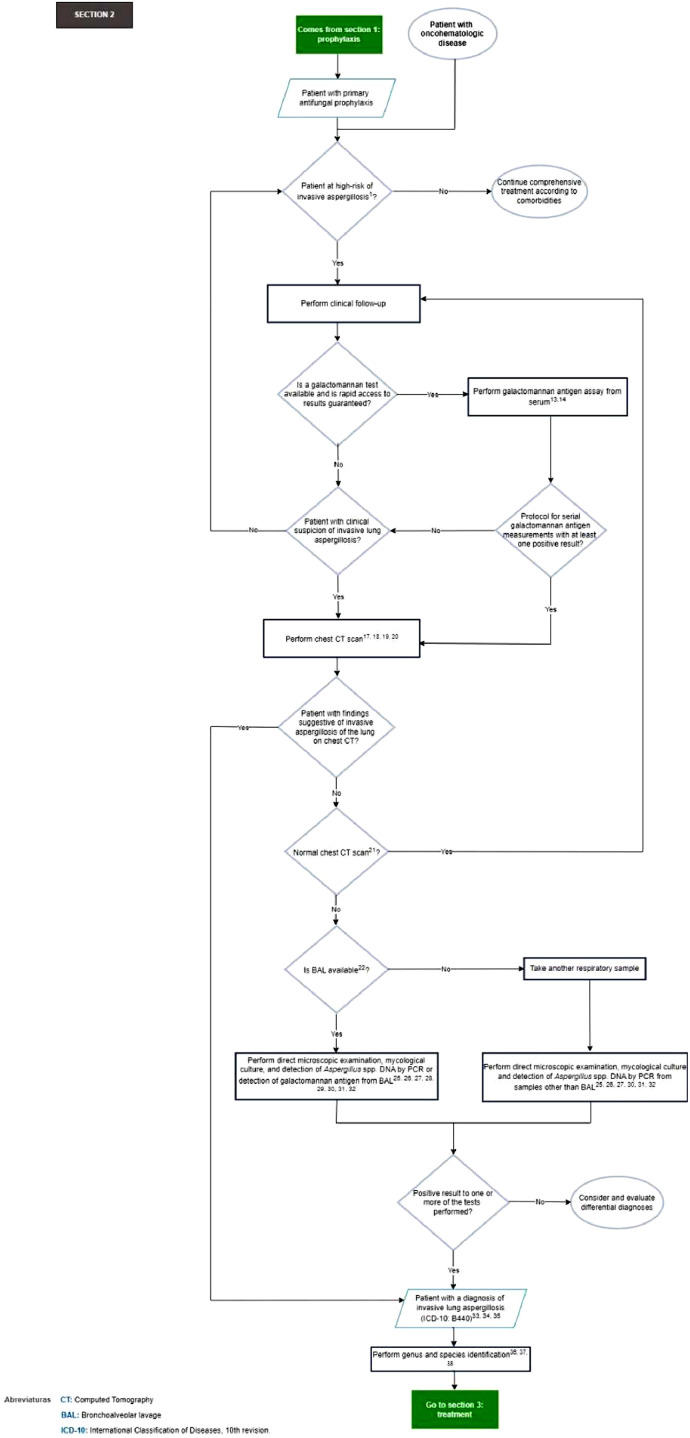
Section 2 of the flowchart: Diagnosis of the patient with oncohematologic disease with clinical suspicion of invasive lung aspergillosis. CT, Computed Tomography; BAL, Bronchoalveolar Lavage; ICD-10, International Classification of Diseases, 10th revision.

In Fig. 3, section 2 of the flowchart (diagnostic approach to the patient with oncohematologic disease with cynical suspicion of invasive lung aspergillosis) is presented.

***Recommendations flowchart Section 2:*** Diagnosis of the patient with oncohematologic disease with cynical suspicion of invasive lung aspergillosis.

13) For patients at high-risk for invasive lung aspergillosis who do not receive primary antifungal prophylaxis against filamentous fungi, serum-based galactomannan antigen testing, within a serial measurement protocol (two or three times per week), is recommended for early detection and/or diagnosis of invasive lung aspergillosis (strong recommendation, high-quality evidence, GRADE).^6^
  For patients at high-risk for invasive lung aspergillosis on primary antifungal prophylaxis against filamentous fungi who show signs suggestive of invasive lung aspergillosis, galactomannan antigen testing from serum is recommended, within a protocol of measurements per clinical indication (2 or 3 consecutive days), for early detection and/or diagnosis of invasive lung aspergillosis (expert recommendation).^31^, ^32^, ^33^, ^34^
14) For patients at high-risk for invasive lung aspergillosis, serum galactomannan antigen testing is recommended for diagnosing invasive lung aspergillosis (strong recommendation, high-quality evidence, GRADE).^6^
15) The expert panel considers that there is insufficient evidence to recommend using 1,3-β-d-glucan, as it has a limited role in ruling out or making the exclusive diagnosis of invasive aspergillosis.^23^
16) For patients at high-risk for invasive lung aspergillosis, tests that detect specific antibodies and/or *Aspergillus* spp. precipitation are not recommended for diagnosing invasive lung aspergillosis (strong recommendation, moderate-quality evidence, GRADE).^6^
17) High-resolution/thin-slice Computed Tomography (CT) of the chest is recommended whenever there is clinical suspicion of invasive lung aspergillosis, regardless of the results of chest radiography (strong recommendation, high-quality evidence, GRADE).^6^^,^^23^
18) Routine use of contrast-enhanced chest CT is not recommended for suspected invasive lung aspergillosis (strong recommendation, high-quality evidence, GRADE).^6^^,^^23^
19) Good practice point: CT pulmonary angiography can improve diagnosis of invasive lung aspergillosis in patients with oncohematologic disease by demonstrating the “vascular occlusion sign”, which is more sensitive than other common CT findings. The search for this sign can be performed in patients with nodular lesions (central > 10 mm or peripheral > 12 mm) or suspected pulmonary infarction, considering the patient's renal function.^35^, ^36^, ^37^
20) For patients at high-risk for invasive lung aspergillosis with clinical findings of hemoptysis, it is recommended to perform arterial phase CT angiography to identify the possible site of vessel erosion (strong recommendation, moderate-quality evidence, GRADE).^6^
21. Good practice point: If the galactomannan antigen test is positive and high-resolution thin-slice/thin-slice CT scan of the chest is normal, consider looking for invasive aspergillosis at other anatomic sites or invasive infection by another filamentous fungus.^38^^,^^39^
22. For suspected invasive lung aspergillosis, bronchoscopy with bronchoalveolar lavage* is recommended (strong recommendation, moderate-quality evidence, GRADE).^6^^,^^23^
* Refractory thrombocytopenia is an absolute contraindication for bronchoalveolar lavage.^40^^,^^41^
23. Good practice point: If there is no response to treatment, atypical findings on chest CT scan, or suspicion of coinfection, consider performing lung biopsy*.^25^^,^^42^
* Severe hypoxemia or severe alterations of hemostasis such as thrombocytopenia refractory to platelet transfusion and thrombocytopenia <50,000 µL are absolute contraindications for lung biopsy.^23^
24. If peripheral nodular lesions are present and if Endobronchial Endoscopic Ultrasound (EBUS) is available, consider using it in lung biopsy (expert recommendation).^23^
25. For patients at high-risk for invasive lung aspergillosis, it is recommended to routinely perform direct microscopic examination and mycological culture of respiratory tract samples (like induced sputum, tracheal aspirates, bronchoalveolar lavage) to recover the possible etiological agent involved in the infectious process (strong recommendation, moderate-quality evidence, GRADE).^6^^,^^25^
26. For patients at high-risk for invasive lung aspergillosis, using optical targets (Calcofluor white™, Uvitex 2B, Blancophor™), or Grocott's Methenamine Silver (GMS) or Periodic Acid-Schiff (PAS) staining during direct microscopic examination of respiratory tract and/or tissue samples from the affected site according to institutional protocols is recommended (strong recommendation, high-quality evidence, GRADE).^6^
27. It is recommended to submit fluid and tissue specimens in adequate quantities for simultaneous histopathological/cytological and microbiological examination (strong recommendation, high-quality evidence, GRADE).^23^
28. For patients at high-risk for invasive lung aspergillosis, consider using lateral flow assay to test for galactomannan antigen in bronchoalveolar lavage as a quick and easy diagnostic tool (conditional recommendation, moderate-quality evidence, GRADE).^6^ The expert panel considers that there is not enough high-quality evidence to favor this method over other techniques for measuring this biomarker.
29. For patients at high-risk for invasive lung aspergillosis, it is recommended to detect *Aspergillus* spp. DNA using PCR from respiratory tract specimens (bronchoalveolar lavage and/or biopsy) and/or whole blood (strong recommendation, moderate-quality evidence, GRADE).^6^
30. Good practice point: In scenarios of isolates with atypical growth or suspected resistance, species identification by molecular methods should be used.^23^
31. Good practice point: For molecular studies, previously validated and implemented laboratory tests should be used.^43^, ^44^, ^45^
32. Good practice point: The results of diagnostic tests must be interpreted within the context of specimen type and the history of antifungal drug use.^25^
33. A joint interpretation of clinical, imaging, microbiology, and pathology criteria is recommended for diagnosing invasive lung aspergillosis (strong recommendation, high-quality evidence, GRADE).^6^
34. It is recommended that invasive lung aspergillosis be considered in patients with the “halo sign” in the high-resolution CT scan of the chest (strong recommendation, moderate-quality evidence, GRADE).^6^
35. In patients at high-risk for invasive lung aspergillosis, isolation of an *Aspergillus* species from respiratory tract specimens (induced sputum, tracheal aspirates, bronchoalveolar lavage) and/or biopsy of the involved site is highly suggestive of invasive lung aspergillosis (strong recommendation, high-quality evidence, GRADE).^6^
36. For patients at high-risk for invasive lung aspergillosis, it is recommended that *Aspergillus* species recovered from respiratory tract specimens (induced sputum, tracheal aspirates, bronchoalveolar lavage) and/or biopsy of the affected site be identified to complex level. All isolates from the *A. fumigati* section/complex should be identified to species level (strong recommendation, high-quality evidence, GRADE).^6^
37. It is recommended to identify *Aspergillus* species involved in invasive aspergillosis by macroscopic and microscopic examination of primary cultures. The use of special identification media (2 % malt extract agar and/or Czapek-Dox agar), incubated at 25 °C–30 °C, 37 °C, and 50 °C (Strong recommendation, moderate-quality evidence, GRADE) is recommended.^6^^,^^25^
38. Consider using proteomic techniques (MALDI-TOF MS, matrix-assisted laser desorption/ionization time-of-flight mass spectrometry), if available, for identifying *Aspergillus* species (conditional recommendation, moderate-quality evidence, GRADE).^25^
39. Antifungal susceptibility testing of Aspergillus isolates during initial infection is not routinely recommended. Performing antifungal susceptibility testing, using a reference method, is reserved for the setting of suspected failure and/or refractoriness to antifungal therapy, or for epidemiological purposes (strong recommendation, moderate-quality evidence, GRADE).^23^

**Fig. 4:**
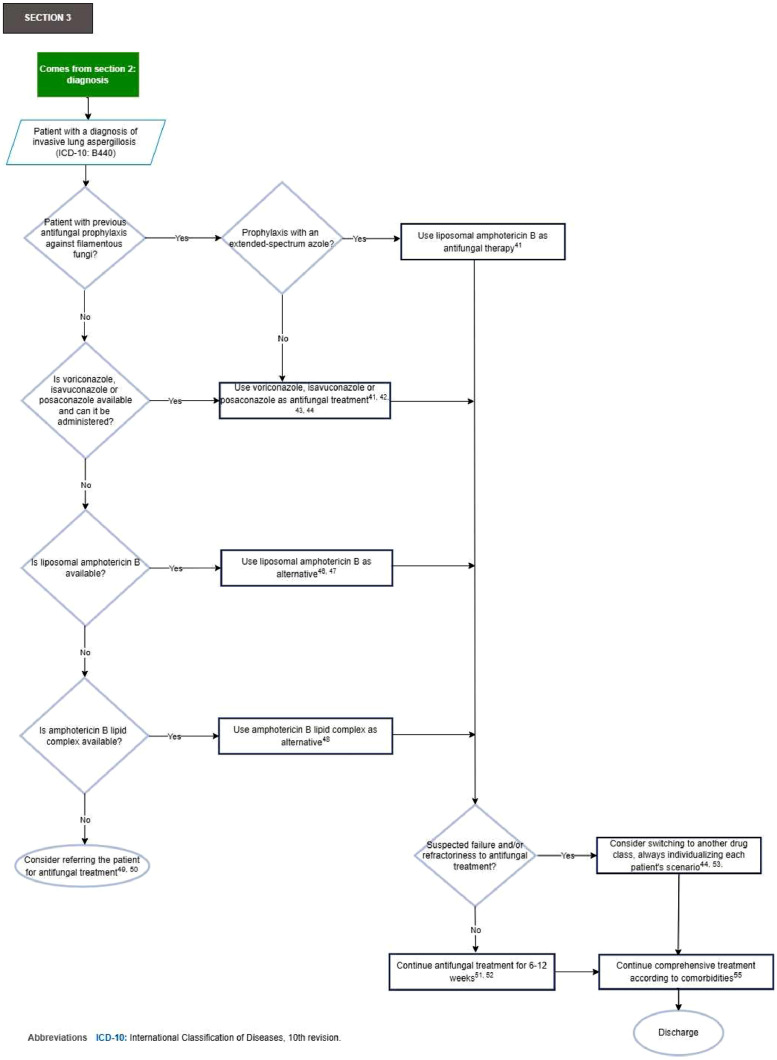
Section 3 of the flowchart: Treatment of the patient with oncohematologic disease diagnosed with invasive lung aspergillosis. ICD-10, International Classification of Diseases, 10th revision.

Fig. 4 shows Section 3 of the flowchart (patient management of the patient with oncohematologic disease diagnosed with invasive lung aspergillosis).

***Recommendations Section 3 of the flowchart:*** Treatment of the patient with oncohematologic disease diagnosed with invasive lung aspergillosis

40. Start empirical antifungal treatment against filamentous fungi for patients with prolonged neutropenia (> 10-days), no prior antifungal prophylaxis against filamentous fungi, with clinical signs/symptoms of invasive lung aspergillosis, and suggestive findings of invasive aspergillosis on chest CT scan, and without availability and/or timely access to clinical laboratory diagnostic tools (strong recommendation, moderate-quality evidence, GRADE).^26^
41. In patients with previous antifungal prophylaxis against filamentous fungi, the choice of antifungal treatment against filamentous fungi is recommended based on the type of prophylaxis administered (strong recommendation, high-quality evidence, GRADE)^25^^,^^26^:
  • If the patient has received prophylaxis with an extended-spectrum azole, liposomal amphotericin B is recommended for antifungal treatment (Table 3).

**Table 3:** Antifungal treatment of patients with oncohematologic disease diagnosed with invasive lung aspergillosis.

**Antifungal**	**Dosage**
Voriconazole	6 mg/kg every 12 h on the 1st day then continue with 4 mg/kg every 12 h IV
	4 mg/kg every 12 h PO
Isavuconazole	200 mg every 8 h for 2 days and then continue 200 mg everyday PO or IV
Posaconazole	300 mg every 12 h on the 1st day then continue with 300 mg everyday Tablet PO or IV
	400 mg every 12 h or 200 mg every 6 h PO Suspension
Liposomal amphotericin B	3 mg/kg everyday IV
Amphotericin B lipid complex	5 mg/kg everyday IV

PO, Per Oral; IV, Intravenous.

Taken and adapted from.^23^^,^^25^.
  • If the patient has received prophylaxis with echinocandin, the use of an extended-spectrum azole as an antifungal treatment is recommended. Consider the use of liposomal amphotericin B as an alternative (Table 3).
42. In patients without previous antifungal prophylaxis against filamentous fungi, the use of an extended-spectrum azole (voriconazole, isavuconazole, or posaconazole) is recommended as an antifungal treatment (strong recommendation, high-quality evidence, GRADE)^23^^,^^26^ (Table 3).
43. Good practice point: When using extended-spectrum azoles as an antifungal treatment for invasive lung aspergillosis, potential drug–drug interactions should be carefully reviewed^23^^,^^25^^,^^46^ (Table 4).

**Table 4:** Main drug interactions to consider in patients with oncohematologic disease under treatment for invasive lung aspergillosis with extended-spectrum azoles.

**Azol**	**Effect on metabolic enzymes and transport proteins**	**Chemotherapeutic agent with interaction**
Voriconazole	Mild inhibitor of CYP2C19, moderate inhibitor of CYP2C9, and strong inhibitor of CYP3A4	Holo-transretinoic acid, axitinib, cyclosporine, ibrutinib, midostaurin, ruxolitinib, arsenic trioxide
Isavuconazole	Moderate inhibitor of CYP3A4/5, and mild inhibitor of gp-P, OCT2 and UGT. Mild CYP2B6 inducer	Bosutinib, cyclophosphamide, venetoclax
Posaconazole	Potent inhibitor of CYP3A4	Acalabrutinib, all-trans retinoic acid, bosutinib, cyclophosphamide, cyclosporine, dasatinib, midostaurin, ruxolitinib, venetoclax

CYP, Cytochrome P450; gp-P, P-glycoprotein; OCT2, Organic Cation Transporter 2; UGT, Uridine Diphosphate Glucuronyltransferase.

Taken and adapted from.^46^^,^^52^.
44. Good practice point: If drug–drug interaction, therapeutic failure, and/or toxicity is suspected, use serum concentration measurements of voriconazole and/or posaconazole, if available, to guide antifungal treatment^23^^,^^25^^,^^46^ (Table 5).

**Table 5:** Measurement of serum concentrations of voriconazole or posaconazole in the patient with oncohematologic disease under treatment for invasive lung aspergillosis.

**Antifungal**	**Moment of measurement**	**Therapeutic goal**	**Toxicity**
Voriconazole	Between the 4th and 7th day of starting treatment or the 4th day after an adjustment is made	Prophylaxis: > 1 mg/dL.	Maintain < 5–6 mg/dL to reduce risk
		Treatment: 1–5.5 mg/dL or Cmin/MIC 2–5	
Posaconazole	Between the 4th and 7th day of starting treatment	Prophylaxis: > 0.7 mg/dL	Maintain < 3.75 mg/dL to reduce risk
		Treatment: > 1 mg/dL	

Cmin, Minimum Concentration/valley; MIC, Minimum Inhibitory Concentration.

Taken and adapted from.^23^^,^^26^^,^^53^.
45. Good practice point: The use of itraconazole, or itraconazole-SUBA as an antifungal treatment for invasive lung aspergillosis is discouraged.^23^
46. Consider using liposomal amphotericin B as an alternative for antifungal treatment, when the use of azoles is contraindicated and/or not possible (strong recommendation, moderate-quality evidence, GRADE)^25^^,^^26^ (Table 3).
47. Good practice point: For patients with renal failure who cannot receive extended-spectrum azole, liposomal amphotericin B can be used as an antifungal treatment, and renal replacement therapy can be initiated.^47^, ^48^, ^49^
48. Use lipid complex amphotericin B as an antifungal treatment if liposomal amphotericin B is contraindicated and/or not possible (Expert recommendation)^26^ (Table 3).
49. The use of amphotericin B deoxycholate as an antifungal treatment is not recommended (expert recommendation).^26^
50. Good practice point: Avoid using echinocandins (caspofungin, anidulafungin or micafungin) in monotherapy as an antifungal treatment if azoles and polyenes are contraindicated and/or not possible.^50^^,^^51^
51. It is recommended that treatment of invasive lung aspergillosis continue for 6–12 weeks, depending largely on the degree and duration of immunosuppression and evidence of disease improvement (strong recommendation, low-quality evidence, GRADE).^23^
52. Consider changing the route of administration of antifungal treatment from intravenous to oral route in clinically stable patients with reliable enteric absorption (expert recommendation).^25^
53. Good practice point: In scenarios of therapeutic failure, consider switching to another class of drug, always individualizing the scenario for each patient.^25^
54. The expert panel considers that there is insufficient high-quality evidence to recommend for or against the use of combination antifungal therapy for treating invasive lung aspergillosis.
55. For patients who have successfully treated invasive lung aspergillosis and will undergo a new period of immunosuppression, it is recommended to initiate secondary antifungal prophylaxis to prevent recurrence (strong recommendation, moderate-quality evidence, GRADE).^23^

### Implementation of EBCS

To ensure safe and quality care for adult patients with oncohematological disease in Latin America, the implementation of this EBCS for the diagnosis and treatment of invasive lung aspergillosis in this population is proposed within the framework of the activities that are part of the optimization programs in using antifungals, considering for its adequate management the measurement of the indicators presented in Table 6, proposed as control points, for their measurement and obligatory reporting with the frequency that each institution considers pertinent according to its daily clinical practice.

**Table 6:** Indicators for the implementation of EBCS.

**Name**	**Definition**	**Numerator**	**Denominator**
1. Use of posaconazole as primary antifungal prophylaxis	Proportion of adult patients with oncohematologic disease and high risk of invasive aspergillosis^a^, using posaconazole as primary antifungal prophylaxis.	Total number of adult patients with oncohematologic disease and high risk of invasive aspergillosis^a^, without contraindication to the use of azoles, with use of posaconazole as primary antifungal prophylaxis.	Total number of adult patients with oncohematologic disease and high risk of invasive aspergillosis^a^, without contraindication to the use of azoles.
2. High-resolution / thin-slice chest CT	Proportion of adult patients with oncohematologic disease diagnosis and invasive lung aspergillosis suspected, with high-resolution / thin-section chest CT.	Total number of adult patients with oncohematologic disease diagnosis and invasive lung aspergillosis suspected, with high-resolution / thin-slice chest CT	Total number of adult patients with oncohematologic disease diagnosis and invasive lung aspergillosis suspected
3. Bronchoscopy with BAL	Proportion of adult patients with oncohematologic disease diagnosis and invasive lung aspergillosis suspected, with bronchoscopy with BAL performed.	Total number of adult patients with oncohematologic disease diagnosis and invasive lung aspergillosis suspected, without contraindication to perform BAL, with bronchoscopy with BAL.	Total number of adult patients with oncohematologic disease diagnosis and invasive lung aspergillosis suspected, without contraindication to perform BAL
4. Direct microscopic examination and mycological culture	Proportion of adult patients with oncohematologic disease diagnosis and invasive lung aspergillosis suspected, with direct microscopic examination and mycological culture of respiratory tract samples (induced sputum, tracheal aspirates, BAL).	Total number of adult patients with oncohematologic disease diagnosis and invasive lung aspergillosis suspected, with direct microscopic examination and mycological culture of respiratory tract samples (induced sputum, tracheal aspirates, BAL).	Total number of adult patients with oncohematologic disease diagnosis and invasive lung aspergillosis suspected
5. Use of an extended-spectrum azole as initial antifungal therapy.	Proportion of adult patients with oncohematologic disease, without previous antifungal prophylaxis against filamentous fungi, and diagnosed with invasive lung aspergillosis, using an extended-spectrum azole (voriconazole, isavuconazole, or posaconazole) as initial antifungal therapy.	Total number of adult patients with oncohematologic disease, without previous antifungal prophylaxis against filamentous fungi, and diagnosed with invasive lung aspergillosis, without contraindication for the use of azoles, with use of an extended-spectrum azole (voriconazole, isavuconazole or posaconazole) as initial antifungal therapy.	Total number of adult patients with oncohematologic disease, without previous antifungal prophylaxis against filamentous fungi, and diagnosed with invasive lung aspergillosis, without contraindication for the use of azoles.

a Patients with severe graft-versus-host disease or acute myeloid leukemia or myelodysplastic syndrome on induction with intensive chemotherapy.

To implement this guide, the following dissemination tools will be used to facilitate its access to health professionals: the publication of the EBCS in The Brazilian Journal of Infectious Diseases, and the inclusion of the algorithms and recommendations in online courses and mobile applications.

### EBCS update

This EBCS should be updated within a maximum of five years, following the methodology and standards that have been established for developing evidence-based recommendations. The topics may be reconsidered according to the need for publication of new evidence.

### Ethical considerations

The regulations outlined in national and international legislation have guided the development of this work, adhering to the ethical and bioethical standards for scientific research.

## Funding source

This document has been developed as part of the “Antifungal administration programs in a Latin American country” project of the Universidad Nacional de Colombia, financed by a grant from Pfizer Inc. The project's objectives included developing recommendations for diagnosis and treatment of invasive lung aspergillosis in the patient with oncohematologic disease in Latin America. In addition, the publication was supported by the School of Medicine of the Universidad Nacional de Colombia, through funding for the edition of the manuscript in English.

## Editorial independence

The content of this document was developed entirely independently, free from the influence of the funding entity, Pfizer Inc., which did not participate or have any role in the different phases of the development of the EBCS, including among others: the formation of the development group; the systematic search, screening, and selection of CPG; the analysis and interpretation of the evidence; the formation of the expert consensus; the generation of recommendations and preparation of the algorithms; the drafting of the manuscript; and the decision to publish. None of the professionals who were part of the development group, or the expert consensus, received any type of payment or incentive for their participation in or contributions to the development of this document.

## Authors’ contribution

JAC conceptualized and led the EBCS. MCV performed the methodological coordination, systematic search of CPGs, and preparation of the comparative evidence table. JAC, MCV, DAR, LCN, and CDB contributed to the screening, quality assessment, selection of CPG, and wrote the first draft of the manuscript and the final version. JAC, MCV, DAR, LCN, CDB, MN, RMR, DC, CAA, FV, and LE participated as members of the development group in defining the scope and objective of the EBCS, and in the process of reviewing, discussing, and drafting the proposed recommendations and preliminary algorithm. SIC, LT, DEC, LEC, EPV, FR, PC, RAR, BLG, AMCR, DLO, JLS, and MS participated as members of the expert consensus in the review, discussion, and final elaboration of recommendations and algorithms. The initial iteration of the manuscript was reviewed by all authors, and the final version was read and approved by all authors.

## Conflicts of interest

The following authors declared no conflict of interest: DAR, DC, DCN, LCN, CD, SIC, LEC, BLG, AMCR, DLO. The following authors declared conflict of interest: JAC (Pfizer), MCV (Pfizer), RMR (Gilead Sciences, Gador, Pfizer), CAA (Moderna, Merck Sharp & Dohme, GlaxoSmithKline, Becton Dickinson, Pfizer), MN (Pfizer, Merck Sharp & Dohme, Knight Therapeutics), LT (Gador, Gilead Sciences, Knight Therapeutics), DEC (IMMY), EPV (Pfizer), FR (Gador, Gilead Sciences, Knight Therapeutics), PC (Pfizer, Merck Sharp & Dohme), RAR (Pfizer, SteinCares, Merck Sharp & Dohme). With no stated interests identified that could be considered potentially conflicting with the primary interest of this EBCS.

## Appendix Supplementary materials

Image, application 1
