# Supplementary material for: Evidence-based clinical standard for the diagnosis and treatment of invasive lung aspergillosis in the patient with oncohematologic disease
Source: Braz J Infect Dis. 2025 Feb 24;29(2):104517. doi: 10.1016/j.bjid.2025.104517 (PMC11903820; doi:10.1016/j.bjid.2025.104517)
Supplement: Supplementary file 1 [file mmc1.docx]

BJID-D-24-00236_ **Supplementary material**

**Evidence-based clinical standard for the diagnosis and treatment of invasive lung aspergillosis in the patient with oncohematologic disease**

**Supplementary Table 1** Full search strategies for databases.

| **MEDLINE search strategy:** | |
| --- | --- |
| 1 | exp Mass Screening/ |
| 2 | screening*.ti,ab. |
| 3 | (screening* adj2 (multiple or program* or mass)).ti,ab. |
| 4 | exp Diagnosis/ |
| 5 | diagnos*.ti,ab. |
| 6 | prophylaxis.ti,ab. |
| 7 | (prophyla* adj2 (therap* or treatment* or management or medication*)).ti,ab. |
| 8 | (pre?emptive adj2 (therap* or treatment* or prophylaxis)).ti,ab. |
| 9 | exp Therapeutics/ |
| 10 | (therap* or treatment* or manag*).ti,ab. |
| 11 | or/1-10 |
| 12 | exp Aspergillus/ |
| 13 | exp Aspergillosis/ |
| 14 | exp Pulmonary Aspergillosis/ |
| 15 | (Aspergill* adj2 (infecio* or fungemia* or invasiv* or Pulmonar* or Lung or Pleural or Bronchopulmonar*)).ti,ab. |
| 16 | (Fung* adj2 (infecio* or invasiv* or Pulmonar* or Lung or Pleural or Bronchopulmonar*)).ti,ab. |
| 17 | fumigatus.ti,ab. |
| 18 | or/12-17 |
| 19 | exp Hematologic Neoplasms/ |
| 20 | ((Hemato* or blood) adj2 (Neoplasm* or Malignan* or Cancer)).ab,ti. |
| 21 | exp Hematopoietic Stem Cell Transplantation/ |
| 22 | (Hematopoietic Stem Cell adj2 (Transplant* or recipient*)).ti,ab. |
| 23 | exp Bone Marrow Transplantation/ |
| 24 | (Bone Marrow adj2 (Transplant* or recipient*)).ti,ab. |
| 25 | or/19-24 |
| 26 | 11 and 18 and 25 |
| 27 | exp practice guidelines as topic/ |
| 28 | exp practice guideline/ |
| 29 | practice guideline.pt. |
| 30 | (practice adj2 guide*).ti,ab. |
| 31 | guideline.pt. |
| 32 | consensus development conference.pt. |
| 33 | recommendation*.ti,ab. |
| 34 | or/27-33 |
| 35 | 26 and 34 |
| 36 | limit 35 to yr="2014 -Current" |
| **EMBASE search strategy:** | |
| 1 | 'screening'/exp |
| 2 | screening* |
| 3 | screening* NEAR/2 (multiple OR program* OR mass) |
| 4 | diagnosis'/exp |
| 5 | diagnos* |
| 6 | 'prophylaxis'/exp |
| 7 | prophyla* NEAR/2 (therap* OR treatment* OR management OR medication*) |
| 8 | 'preemptive therapy'/exp |
| 9 | pre?mptive NEAR/2 (therap* OR treatment* OR prophylaxis) |
| 10 | therapy'/exp |
| 11 | therap* OR treatment* OR manag* |
| 12 | OR/1-11 |
| 13 | 'aspergillus'/exp |
| 14 | 'aspergillosis'/exp |
| 15 | 'lung aspergillosis'/exp |
| 16 | aspergill* NEAR/2 (infecio* OR fungemia* OR invasiv* OR pulmonar* OR lung OR pleural* OR bronchopulmonar*) |
| 17 | fung* NEAR/2 (infecio* OR invasiv* OR pulmonar* OR lung OR pleural* OR bronchopulmonar*) |
| 18 | 'fumigatus':ab,ti |
| 19 | OR/13-18 |
| 20 | hematologic malignancy'/exp |
| 21 | (hemato* OR blood) NEAR/2 (neoplasm* OR malignan* OR cancer) |
| 22 | hematopoietic stem cell transplantation'/exp |
| 23 | 'hematopoietic stem cell' NEAR/2 (transplant* OR recipient*) |
| 24 | 'bone marrow transplantation'/exp |
| 25 | bone marrow' NEAR/2 (transplant* OR recipient*) |
| 26 | OR/20-25 |
| 27 | #12 AND #19 AND #26 |
| 28 | practice guideline'/exp |
| 29 | practice* NEAR/2 guide* |
| 30 | guideline* |
| 31 | consensus NEAR/2 development NEAR/2 conference |
| 32 | recommendation* |
| 33 | OR/28-32 |
| 34 | #27 AND #33 |
| 35 | #34 AND (2014:py OR 2015:py OR 2016:py OR 2017:py OR 2018:py OR 2019:py OR 2020:py OR 2021:py OR 2022:py OR 2023:py) |

**Supplementary Figure 1** PRISMA flowchart.
